# Influence of Carbohydrate Intake on Caprylic Acid (C8:0)-Induced Ketogenesis—A Systematic Review and Meta-Analysis

**DOI:** 10.3390/nu16152456

**Published:** 2024-07-29

**Authors:** Marius Frenser, Tobias Fischer, Isabel Albrecht, Thorsten Marquardt

**Affiliations:** 1 Department of Food, Nutrition, and Facilities, FH Muenster, University of Applied Sciences Muenster, 48149 Muenster, Germany; 2 Department of General Pediatrics, Metabolic Diseases, University Hospital Muenster, 48149 Muenster, Germany

**Keywords:** ketones, ketone bodies, metabolism, caprylic acid (C8; C8:0), ß-hydroxybutyrate, medium-chain fatty acids (MCFA), medium-chain triglycerides (MCT), ketogenic diet, carbohydrates

## Abstract

The ketogenic diet is used worldwide to treat various diseases, especially drug-resistant epilepsies. Medium-chain triglycerides or medium-chain fatty acids, primarily the major ketogenic compound caprylic acid (C8; C8:0), can significantly support ketogenesis. This review examines the effects of concurrent carbohydrate intake on C8-induced ketogenesis. A systematic literature search (PubMed and Web of Science) with subsequent data extraction was performed according to PRISMA guidelines and the Cochrane Handbook. Studies investigating the metabolic response to C8-containing MCT interventions with carbohydrate intake were included. The studies did not include a ketogenic diet. Three intervention groups were created. The quality of the studies was assessed using the RoB II tool, and the meta-analysis was performed using the Cochrane RevMan software. A total of 7 trials, including 4 RCTs, met the inclusion criteria. Ketone production was lower when C8 was combined with carbohydrates compared to MCT intake alone. The lower C8 dose group (11 g) did not show a significantly lower ketogenic effect than the higher dose group (19 g). Forest plot analysis showed heterogeneous data. The data suggest a non-linear relationship between C8, carbohydrate intake and ketone production. Further studies are needed to investigate the influence of different carbohydrates on C8-induced ketogenesis. Limitations include heterogeneous intervention conditions, such as different types of dispersions, caffeine intake, limited number of studies and variability in study design.

## 1. Introduction

When carbohydrate (CH) availability is limited and, insulin levels are low, glycogen stores are depleted, and fatty acids are increasingly used for energy. Acetyl-CoA is produced by ß-oxidation, and its excess is converted to acetoacetate (AcAc) and ß-hydroxybutyrate (ßHB) in hepatic ketogenesis. A small amount of acetone is also formed by irreversible spontaneous decarboxylation. The molecules AcAc, ßHB (chemically: hydroxycarboxylic acid) and acetone are referred to as ketone bodies (ketones) in the human organism [1,2,3]. The ketogenic diet (KD), which aims to produce ketones or induce ketosis [4], was developed in 1921 by Dr Wilder at the Mayo Clinic in Rochester, Minnesota, for children with epilepsy [5]. It is considered more effective than many anticonvulsants [6] and has been used to treat epilepsy for decades. The exact mechanism of action is still unknown [3]. KD is gaining popularity among scientists and the general public worldwide [7,8,9]. In addition to the classic KD with a fat-to-non-fat ratio (ketogenic ratio) of 3:1 or 4:1, less restrictive and newer variants such as the modified Atkins diet (MAD) and low glycaemic index therapy (LGIT) are used in clinical practice [10,11,12]. In very restrictive forms, such as 4:1, about 90% (weight/weight) of the macronutrients are fats [9]. Furthermore, they often contain less than 50 g of CH per day and moderate amounts of protein, whereby the daily limit of 50 g of CH is based on the 1999 International Dietary Energy Consultancy Group (IDECG) [4]. KDs are often associated with side effects, particularly gastrointestinal complaints, such as constipation, vomiting, abdominal pain and diarrhoea. They are also linked to hyperlipidemia, the occurrence of renal calculi, pancreatitis and cardiac abnormalities and are often discontinued after a brief time [13,14]. According to a systematic review that analysed 22 studies on adherence to KD in epilepsy in different age groups, the average compliance rate after one year is 66.7%, and after three years, only 37.7% [15]. Complicating factors include preparation effort, preparation difficulties and dietary restrictions [15,16]. Another form, the MCT-based ketogenic diet (MCTKD), first described by Huttenlocher in 1976, is known to be easier to follow [17]. When the MCTs were initially described, it was noted that they lead to ketosis more quickly, allowing for a higher CH intake. This allows for greater flexibility and a wider range of food choices, which in turn improves the feasibility of the KD [17,18,19]. MCTs or medium-chain fatty acids (MCFAs), which include caproic acid (C6; C6:0), caprylic acid (C8; C8:0), capric acid (C10; C10:0) and, depending on the literature source, lauric acid (C12; C12:0), have been demonstrated to promote ketogenesis due to rapid absorption via the portal vein, carnitine-independent transport into the mitochondria and the resulting faster oxidation to acetyl-CoA in the liver.

Commercially available MCT products typically contain C8, C10 and occasionally C12 [20,21,22,23,24,25,26]. In their natural state, MCFAs are found in greater quantities in coconut and palm kernel oil (coconut: 5–10% C8, 5–8% C10, 45–54% C12; palm kernel: 2–6% C8, 3–7% C10, 51–55% C12) and in smaller amounts in milk fat [27,28,29]. The efficacy of MCTKD is considered to be comparable to that of the classic KD [30]. The original variant had a fat content of 72 En%, of which 60 En% was MCT, with a simultaneous intake of 18 En% CH and 10 En% protein. MCTs provide approximately 8.3 to 8.4 kcal per gram and are an immediate source of energy for the body due to their rapid metabolisation [27,31]. MCTs can enhance the tolerability of low-carbohydrate diets [32], although it is essential to introduce them gradually to prevent gastrointestinal side effects, such as diarrhoea, nausea, cramps and flatulence [18]. The ketogenic effect of C8 is approximately three times higher than that of C10 and approximately six times higher than that of C12 [33]. In an animal model, C6 has been shown to have an even greater ketogenic effect than C8, but it has not been widely used in humans to date [34]. While C8 is known to promote ketogenesis, C10 is suggested to support glycolysis [35]. Following hydrolysis and absorption, MCTs are not re-esterified to triglycerides; therefore, they are less frequently used as storage fat [28].

The difficulties in the feasibility of the KD and the high dropout rates could be reduced by a higher CH intake in the total diet using a C8-containing MCTKD, as this would facilitate everyday life. In this context, it is necessary to clarify the effects of CH intake in combination with C8 on the production of ketones and how different amounts of CH and C8 intake affect ketogenesis. In this context, a systematic literature review on the effect of CH on C8-induced ketogenesis after overnight fasting was conducted to provide a basis for further research questions on factors influencing ketogenesis.

## 2. Materials and Methods

The systematic literature review was conducted in accordance with the methodological standards of the PRISMA guideline (Preferred Reporting Items for Systematic Reviews and Meta-Analyses) [36], taking into account the methodological recommendations of the Cochrane Handbook [37].

### 2.1. Search Strategy, Selection Criteria and Screening

A comprehensive literature search was performed between October and November 2023 in the electronic databases PubMed and Web of Science to identify relevant literature. A sensitive search strategy was created with the help of selected keywords and terms and the use of appropriate operators to cover as much of the relevant literature as possible. A search was conducted for the three main categories of interest: “carbohydrate intake”, “medium-chain triglycerides” and “ketogenesis”. These categories were linked together using the Boolean operator “AND”. In order to supplement the categories, corresponding keywords and mesh terms (PubMed) were also included. To refine the search, the filters “English language” and “human studies” were selected, excluding reviews and meta-analyses. The precise search query for the two databases is presented in Appendix A.

The criteria used to include or exclude studies in the present study are presented in Table 1, based on the PICO scheme (population, intervention, comparison and outcomes) [38].

In accordance with the recommendations of the Cochrane Handbook [39], the results of the study search were imported into the literature management programme Citavi (version 6.17). Duplicates were removed. The titles and abstracts of the remaining studies were then checked for the criteria mentioned (Table 1), and all full texts of the study selection were finally obtained. In addition, further sources were searched from the reference lists of the identified literature and expert suggestions to supplement the present selection. The search results were reviewed by two independent nutritional scientists.

### 2.2. Data Extraction

The following data were extracted in Microsoft Excel (Microsoft Office Professional Plus 2016, version 1808) according to the recommendations of the Cochrane Handbook [40]: general information (authors, title, location and year), study design (study type, randomisation and blinding), participants (population, number, age, sex, dropout, physical and health conditions), intervention (meal and MCT composition, dose/quantity, intervention time and framing conditions), statistical analysis methods, measurement parameters and results, limitations and conflicts of interest/funding. The exact measurement results (means, medians and standard deviations (SD) of plasma glucose, plasma-ßHB, breath acetone and AUC) of the studies were requested from the authors in advance by e-mail in case of unclear or insufficient information. If there was insufficient feedback, the values of the results were determined from the published diagrams and converted to the appropriate values according to the specified scaling. If the exact value of the SD could not be read due to graphical overlaps, a measurement range was specified as “≤ value”, and the maximum value of the SD was assumed during data processing. The graphical analyses of the data were then checked by another scientist. For minor discrepancies in measurements, the mean value of the measurements was calculated; for larger differences, the source of the error was identified, and a new measurement was conducted. As the studies by St-Pierre et al. (2019) [21], Vandenberghe et al. (2017) [24] and Courchesne-Loyer et al. (2017) [25] stated the increase vs. t_0_ (baseline) instead of the absolute measured values, the measurement data of the baseline measurement (t_0_) were added to the stated measured values to ensure uniform data collection. Due to a lack of baseline data in St-Pierre et al. (2019) [21], no adjustments could be made in this study.

The focus was on those interventions in which MCT containing C8 was supplied in combination with a meal or with CH. Furthermore, the interventions had to be assignable to the groups described in Section 2.3. Those arms whose C8 dose was quantifiable were considered. Study arms in which coconut oil was used were excluded due to the fluctuating or unclear C8 content and the high C12 content (approx. 45–54%) [27,28,29]. In addition, the lack of a ketogenic effect of coconut oil has already been investigated [20].

### 2.3. Data Synthesis

The study data yielded the following result categories: ‘ßHB/total ketones’, ‘AUC ßHB/total ketones’, ‘plasma glucose’ and ‘octanoate (C8) and decanoate (C10)’. In order to standardise the MCT intake amount, the data from the studies by Vandenberghe et al. (2017) [24] and St-Pierre et al. (2019) [21] were converted from volumes (mL) to weights (g) based on their density (MCT oils: ρ ~ 0.95 g/mL [41]). The nutrient composition of the interventions, comprising energy (in kilocalories), protein, CH and fat, was calculated using the EBISpro software (University of Hohenheim, version 2016). An energy content of 8.3 g was calculated for the use of MCTs. Existing nutrient information from the studies was utilised. Standardised units of measurement were defined for the collected data, and the values were adjusted accordingly. The following variables were considered: ßHB or total ketones (ßHB and AcAc) in mmol/L, AUC ßHB or total ketones in [µmol ∗ h]/L, plasma glucose in mmol/L and C8/C10 in µmol/L. As some studies only reported the total ketones (ßHB + AcAc), the total ketones of some studies were calculated in the Results section for better comparability. In addition, the side effects of the MCT interventions reported in the studies were recorded.

Following a comprehensive evaluation of the nutritional value of the various interventions, the following three groups were identified and classified according to their C8 and CH intake levels. These groups were derived from the different intervention arms of the seven studies:

- Group 1: ⌀ 22.5 ± 4.1 g C8 and 1.4 ± 1.2 g CH [20,22,42];
- Group 2: ⌀ 11.4 ± 1.0 g C8 and 61.1 ± 6.1 g CH [21,24,25,43];
- Group 3: ⌀ 18.6 ± 0.9 g C8 and 53.3 ± 18.8 g CH [20,21,24,25,42].

Simplified, this results in groups 1: “23 g C8 without CH”, 2: “11 g C8 + 61 g CH” and 3: “19 g C8 + 53 g CH”. The groups were analysed for the aforementioned result categories.

### 2.4. Quality Assessment

All studies were evaluated using the “Revised Cochrane Risk-of-Bias Tool for Randomised Trials”, or “RoB II” for short, in order to assess the degree of bias [44]. All assessment criteria for the available RCTs [20,21,24,25] were considered in the analysis. In order to ensure a uniform quality assessment, the “RoB II” tool was also used for the non-randomised intervention studies [22,42,43], whereby the assessment category “bias arising from the randomisation process” was adapted to “bias arising from the group formation”. A lack of randomisation in non-randomised studies was not automatically considered a negative or “high risk” factor if the study design did not allow for randomisation, such as grouping by age. The results of the analysis were presented graphically in a separate adaptation of the Rob-Vis tool [45]. According to the RoB II guidance, the overall risk was classified as ‘low risk of bias’ if all the assessment criteria were classified as ‘low risk’. The overall risk was classified as ‘some concerns’ if at least one criterion was rated as ‘some concerns’, but no assessment as ‘high risk’ was available. The overall risk was assessed as ‘high risk’ if at least one domain was assessed as ‘high risk’ or if several domains were rated ‘some concerns’, resulting in an overall significant quality deficit [46].

Furthermore, the studies were analysed for conflicts of interest by assessing the respective risk on the basis of three risk levels (low: no conflict of interest, moderate: financial support or cooperation by/with a company and high: complete study funding by a company or company as the employer). The information provided by the authors as part of the publication was incorporated into the analysis.

### 2.5. Meta-Analysis

Forest plots were created using the online tool “Cochrane RevMan” to analyse the influence of CH or a meal using C8 or MCT on ketogenesis. The test for heterogeneity was programme-controlled using Chi^2^ and I^2^. The determination of the overall effect was based on the z-test. The settings were identical for all analyses (data source: manual/data type: continuous/statistical method: inverse variance/effect measure: mean difference/analysis model: fixed effect/totals: totals and subtotals/test for subgroup differences/study confidence interval: 95%/total confidence interval: 95%). The subject numbers and results (total ketones, calculated in Heidt et al. (2023) [42]), including standard deviations at the start of the study and after 0.5, 2 and 4 h, were used to create the forest plots. Due to differences in measurement parameters and interventions, not all studies provided data at all data collection times, so only the available study data were considered in the forest plots.

## 3. Results

### 3.1. Study Selection

**Figure 1:**
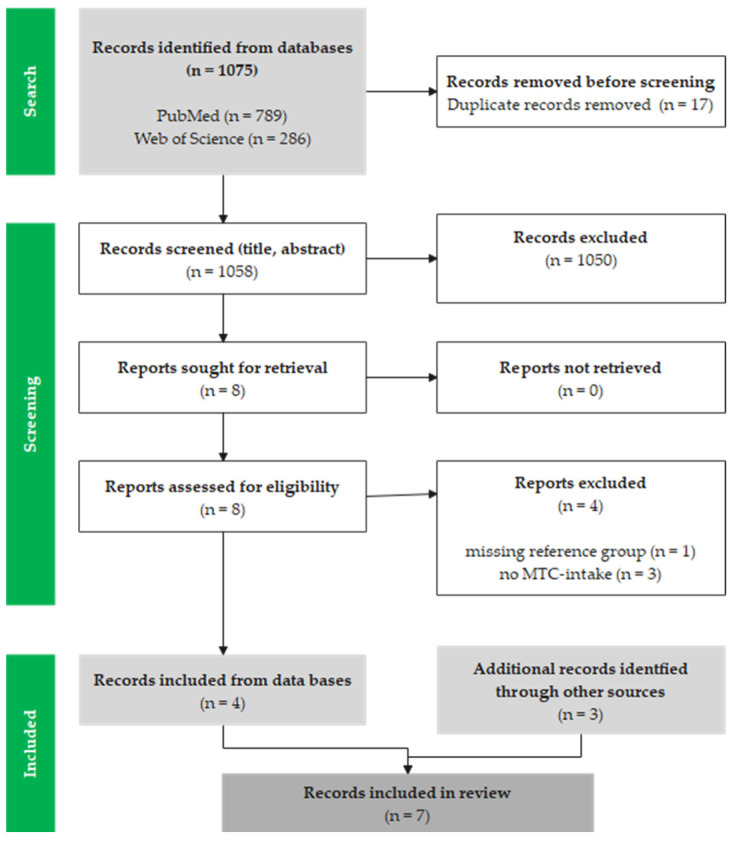
Modified flow chart of the literature search and selection based on the PRISMA flow diagram for systematic reviews [36].

A search in the PubMed and Web of Science databases yielded a total of 1075 results (PubMed: 789 articles; Web of Science: 286 articles). After removing duplicates (n = 17), the titles and abstracts were screened, and a further 1050 studies were removed due to inappropriate thematic focus. After checking the corresponding full texts of eight trials, four further studies were removed, and four studies were included in the review. Three additional studies were supplemented by reviews and citations of other studies so that a total of seven studies could be included in this review (see Figure 1).

### 3.2. Characteristics of the Studies

The main characteristics of the studies are presented in Table 2. The studies were published between 2009 and 2023, with six [20,21,24,25,42,43] of the seven studies published since 2017. A total of 71% of the studies were conducted in Canada [21,22,24,25,43], with further surveys performed in Sweden [20] and Germany [42]. All studies were intervention studies, with six studies (86%) including a control group [20,21,24,25,42,43], four of the seven studies (57%) being randomised or explicitly stating this characteristic [20,21,24,25,42] and five studies (71%) being single-blind [20,21,24,25,42]. The majority of the studies (n = 5; 71%) used a cross-over design [20,21,24,25,42]. In two studies, one intervention was administered per week [20,42], or the time between interventions was at least three days [21,25]. In two further studies, there was no information on a wash-out phase [24,43], although the subjects took part in several study days in each case. In the study by Freemantle et al. (2009), there was no wash-out phase due to the study design with one intervention per group [22]. A total of 114 subjects were examined across the studies, with the number of subjects per study ranging from a minimum of 9 to a maximum of 32 (mean: 15 ± 8). As one study did not provide information regarding the gender of the test subjects [22,24], it is not possible to give an exact indication of the gender distribution. In the remaining studies, the proportion of male and female participants was approximately equal. The average age of participants in four studies was between 25 and 34 years [21,24,25,42], while in one study, the average age was 69 years [20]. Two studies also analysed different age groups from young to old (23–76 years) [22,43]. The inclusion and exclusion criteria varied between the studies depending on the main topic. Diabetics were excluded in all studies, and smokers in almost all studies (exception: Heidt et al. (2023) [42]). Furthermore, a healthy general condition was emphasised across all studies. In four of the seven studies, people who performed high-intensity physical activity or strenuous aerobic exercise >3 times a week and people with coconut or sunflower oil intolerance were excluded [20,21,24,43].

In all studies, the subjects appeared uniformly on the study days after 12 h of overnight fasting. Alcohol on the previous day or within the previous 24 h was prohibited in five studies [20,21,24,42,43]. None of the trials gave specific guidelines on diet before the study days, except for the overnight fast. The subjects consistently appeared between 7:30 and 8:00 AM on the study days. In all studies, a forearm venous catheter was inserted at the beginning, a baseline blood sample was taken, and venous blood was analysed in the laboratory. An exception to the laboratory measurement is the survey by Norgren et al. (2020), in which the ketones were determined from the venous whole blood using a point-of-care measuring device (Statstrip Xpress^®^). According to Norgren et al., the results were validated using a laboratory for individual measurement data, which demonstrated a high correlation [20]. The duration of the interventions was between 4 and 8 h (mean value: 5.7 ± 1.4). Water was freely available during the measurements in all studies; meals outside the planned interventions were prohibited in each case. In three studies [20,22,25], subjects were instructed to minimise physical activity on the study day or to adopt a resting position. The remaining studies did not specify exercise.

### 3.3. Interventions and Controls

**Figure 2:**
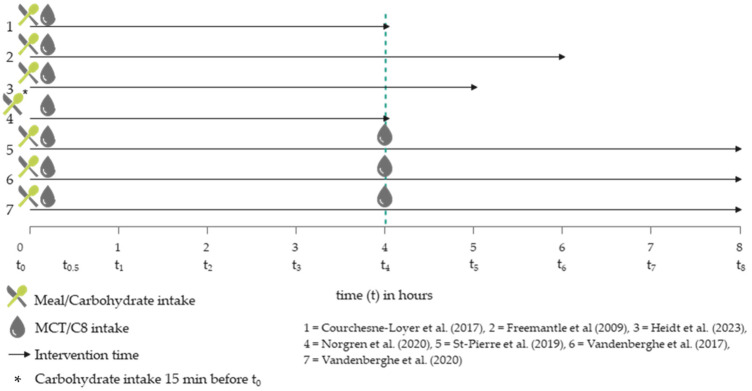
Temporal comparison of MCT and meal interventions in the included trials [20,21,22,24,25,42,43].

The meals were provided at the beginning of the intervention, after the baseline measurement. An exception is the survey by Norgren et al. (2020) [20], in which glucose (50 g in a glass of water) was added 15 min before the baseline measurement to interrupt the fasting state and the associated ketosis. The MCT intake was also uniform in the surveys at the beginning of the study, whereby in three surveys [21,24,43], a further MCT intervention took place after 4 h. Figure 2 compares the described meal and MCT or C8 interventions over time.

Common to all studies was that the test subjects were given CH in the form of glucose, a meal or a drink, as well as MCT containing C8. A total of five studies (71%) aimed to investigate the metabolic response to different MCT interventions in combination with meal(s)/glucose [20,21,24,42,43]. The remaining two studies focused on the response of different age groups to a ketogenic breakfast drink [22] or the response to MCTs in the form of an emulsion (EM) or non-emulsion (NE) [25]. In three studies, a breakfast drink was administered as a meal, which consisted of cream, milk protein, water, MCT, coffee, sunflower oil, coconut oil, glucose and/or water, depending on the study [20,22,42]. In the remaining four studies (57%), a standard breakfast was administered, comprising two slices of toast, jam, cheese and scrambled eggs across all studies. However, in the studies where breakfast was the starting point, only the breakfast of Courchesne-Loyer et al. (2017) did not contain scrambled eggs [21,24,25,43]. The six studies with a control group employed disparate controls. Two studies utilised lactose-free, low-fat milk as a control [21,24], while another two employed a meal without MCT intake as a control [25,43]. Sunflower oil [20] and water [42] were employed as controls on a single occasion each. The precise times of intake and the composition of the interventions, including the quantities of CH and MCTs consumed in the relevant study arms, are presented in Table 3.

In all studies, the MCT was processed in the form of a mixture or an emulsion (see Table 4). St-Pierre et al. (2019) [21], Vandenberghe et al. (2017) [24] and Courchesne-Loyer et al. (2017) [25] emulsified by using a mixer or high-pressure homogeniser (kitchen blender “Magic Bullet^®^” [21,24] and Dairy Products Pilot Plant, Institute of Nutrition and Functional Foods, Université Laval: 2000 pounds/square inch; approx. 0.7 µm mean MCT particle diameter [25]), emulsifiers (E472c (citroglyceride), E471(mono-/diglyerides) [23]) were used in the intervention by Vandenberghe et al. (2020) [43]. No information was provided on the emulsification time [21,24,25]. The mixtures were prepared manually [25,42] or without further specification [20]. Freemantle et al. (2009) [24] did not provide any information on the degree of mixing, so mixing is assumed based on the designation of a “drink”.

The groups mentioned in Section 2.3 were formed from the different study arms. A characterisation of their composition and CH and MCT content is given in Appendix B.

### 3.4. Risk of Bias Assessments

**Figure 3:**
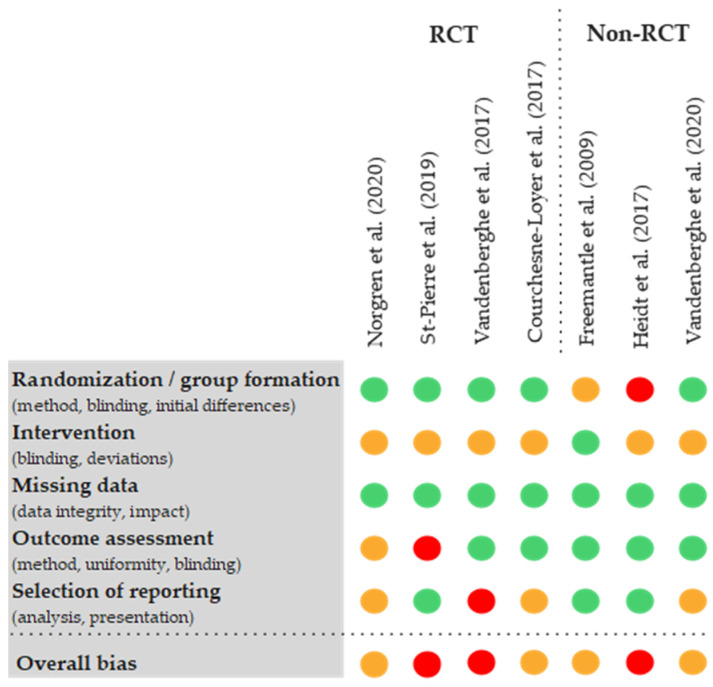
Modified visualisation of the results of the risk of bias assessment using RoB II tools of the available studies [20,21,22,24,25,42,43,45].

The assessment of risk of bias showed limitations in most of the studies. Three studies were classified as ‘high risk’ in the overall conclusion (‘overall bias’). A risk of bias present in three studies was the reporting of total ketones without separate breakdowns of ßHB and AcAc [24,25,43]. In addition, all studies were at most single-blinded. Three of the seven trials were not RCTs, although randomisation would have been possible in one trial [42]. In another study, the control values were subtracted from the measurement data without reporting the actual values or the control value [21]. Another serious limitation was the presentation of results of only selected interventions [24]. The availability and transparency of the data were mostly rated positively. In five of the seven studies, the complete measurement data of all study arms were available [21,22,25,42,43], but an overall problem was an insufficient explanation of the methods used by the authors (see Figure 3).

### 3.5. Conflicts of Interest

The authors of four of the seven studies (57%) reported that they had no commercial or financial conflicts of interest (low risk) [20,22,24,25]. Two studies had a moderate to high risk because one author received research funding and materials and ran his own company related to the topic [21,43]. This author was involved in a total of five of the seven studies, with the remaining three studies having been previously published and no conflicts of interest declared. In one study, there was a high risk of a conflict of interest due to the fact that the author held a research position with a company associated with the product during the conduct of the study and the associated funding of the study [42].

### 3.6. Effect of Interventions

#### 3.6.1. ßHB and Total Ketones

**Figure 4:**
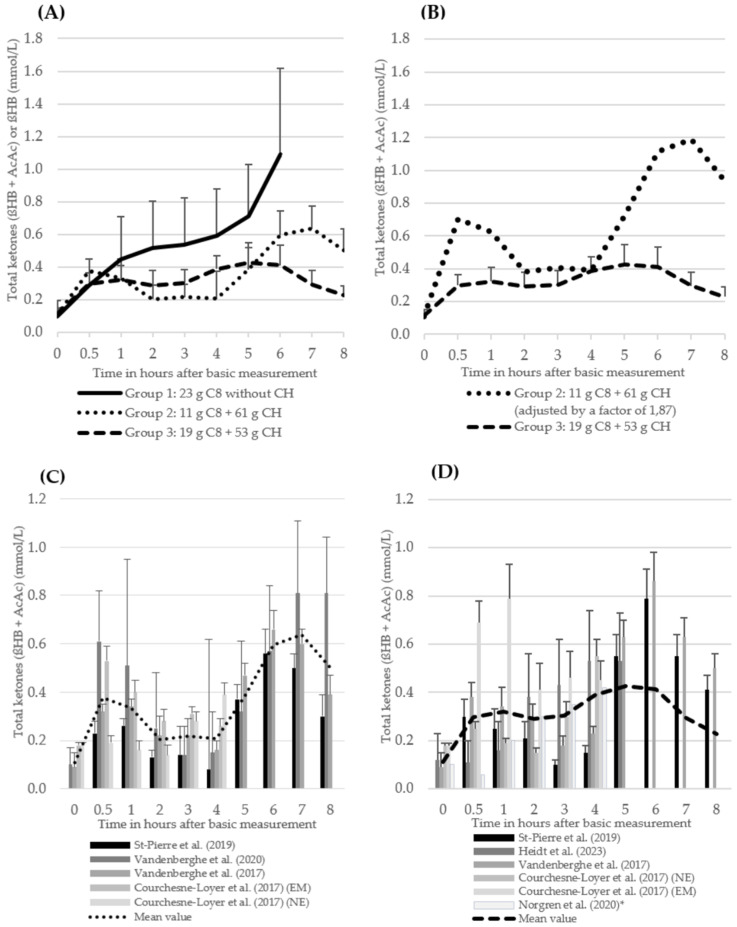
The course of total ketones (ßHB + AcAc) (mean values + standard deviation, only positive values shown here) (**A**) (groups 2 and 3) and of ßHB (group 1) of the three intervention groups. The number of studies (n) of the individual measurement points: group 1: t_0_ = 3, t_0,5_ = 3, t_1_ = 3, t_2_ = 3, t_3_ = 3, t_4_ = 3, t_5_ = 2, t_6_ = 1; group 2: t_0_ = 4, t_0,5_ = 4, t_1_ = 4, t_2_ = 4, t_3_ = 4, t_4_ = 4, t_5_ = 3, t_6_ = 3, t_7_ = 3, T_8_ = 3; group 3: t_0_ = 6, t_0,5_ = 6, t_1_ = 6, t_2_ = 6, t_3_ = 6, t_4_ = 6, t_5_ = 3, t_6_ = 2, t_7_ = 2, T_8_ = 2 (**B**) of group 2 (values multiplied by a factor of 1.87 to achieve a uniform kcal/MCT density of 5.36 between groups 2 and 3) and of group 3 (**C**) of the individual studies from group 2 (**D**) of the individual studies from group 3 ^1^ [20,21,22,24,25,42,43]. ^1^ Norgren et al. (2020) [20]: ßHB instead of total ketones.

The ßHB values of the MCT group without CH intake (group 1) increased continuously over a period of 6 h, with a baseline value of 0.10 ± 0.11 mmol/L, a value of 0.59 ± 0.21 mmol/L at t_4_ and a maximum of 1.09 ± 0.60 mmol/L at t_6_. Total ketones in group 2, which consumed approximately 11 g of C8 and 61 g of CH, started at a baseline value of 0.11 ± 0.03 mmol/L and increased to 0.38 ± 0.08 mmol/L within the first 30 min after the intervention. Subsequently, total ketones decreased to 0.20 ± 0.09 mmol/L (t_2_) and remained constant until t_4_. After the second MCT intervention, total ketones increased again, reaching a maximum value of 0.64 ± 0.14 mmol/L after 7 h. Total ketones in group 3 also increased slightly to 0.30 ± 0.07 mmol/L after 30 min, then reached a plateau and showed a further slight increase at t_4_ (0.39 ± 0.08). After the second MCT intervention (t_4_), total ketones initially remained constant, with an incipient decrease after 6 h to a value of 0.23 ± 0.06 mmol/L at the end of the study (t_8_). Data from three to six studies were available for all values in the period up to t_4_. After t_4_, the database halved to between one and three studies (see Figure 4). The underlying studies of group 1 are uniformly mixtures [20,22,42]. Only one study in group 1 and group 3 included caffeine intake (approximately 170 mg) as part of the intervention [20]. In group 2, with the exception of one intervention arm in Courchesne-Loyer et al. (2017) [25], the MCT was administered as an emulsion in all studies (see Table 4). In group 3, with the exception of Norgren et al. (2020) (ßHB only), total ketones (ßHB + AcAc) were reported or could be calculated in all studies.

**Figure 5:**
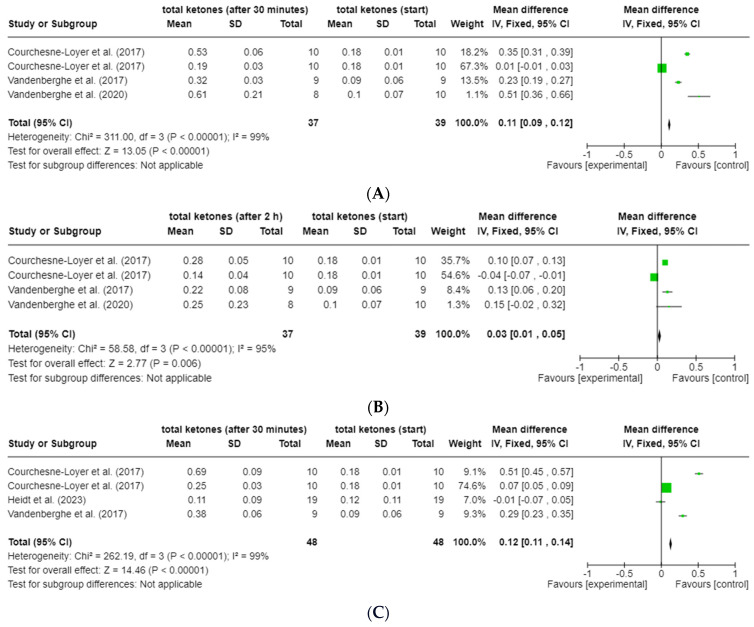

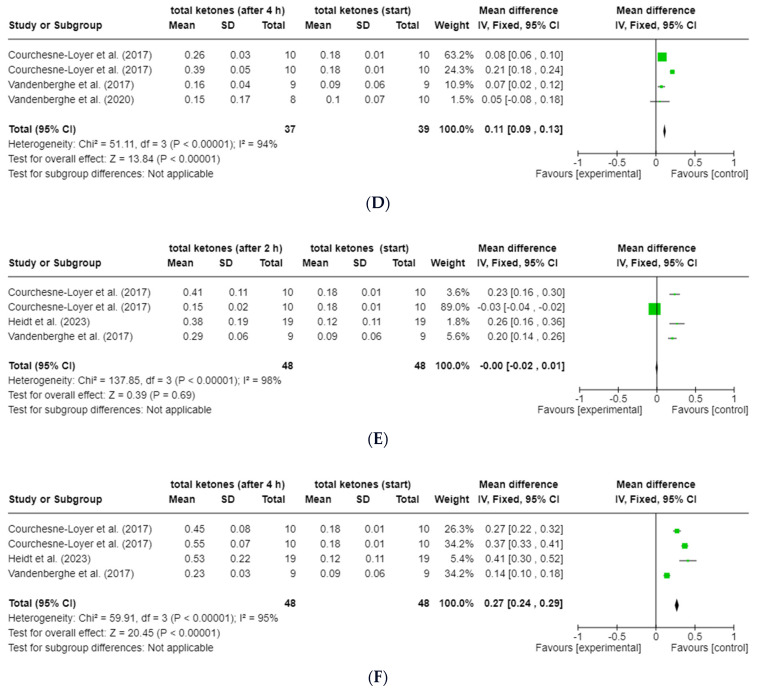
Forest plots for the change in total ketones (ßHB and AcAc) compared to t_0_ after (**A**) 30 min (t_0.5_; group 2), (**B**) 120 min (t_2_; group 2), (**C**) 240 min (t_4_; group 2), (**D**) 30 min (t_0.5_; group 3), (**E**) 120 min (t_2_; group 3) and (**F**) 240 min (t_4_; group 3) [24,25,42,43]. Figure created with Cochrane RevMan software (https://revman.cochrane.org/info accessed on 21 February 2024).

The forest plot analyses of the change in total ketones (ßHB + AcAc) for the time points 30 min (t_0.5_), 120 min (t_2_) and 240 min (t_4_) after the start of the intervention compared to t_0_ for groups 2 and 3 show a very heterogeneous data situation (I^2^ = 94–99%; *p* < 0.00001, Z = 0.39–20.45) between the individual measurement points [47] (see Figure 5a–f). All study data of the present studies from groups 2 and 3 were included, regardless of possible caffeine intake and degree of dispersion (Table 4). The data from the study by St-Pierre et al. (2019) [21] were excluded from this analysis, as no absolute values (actual values) were available due to the lack of baseline data for total ketones. Data from the study by Norgren et al. (2020) [20] could also not be included due to missing SD.

#### 3.6.2. Plasma Glucose

In the MCT group without CH intake (group 1), blood glucose levels were constant over the study period up to t_6_, with values of 4.96 ± 0.42–5.44 ± 0.57 mmol/L [22], 4.80–5.25 mmol/L [20] and 3.72 ± 0.45–4.15 ± 0.46 mmol/L [42]. In group 2, blood glucose levels were also constant up to t_8_, ranging from 4.13 ± 0.50 to 4.68 ± 0.48 mmol/L [43], according to the results of one study. Group 3 showed no particular abnormalities with plasma glucose values of 4.35–5.90 mmol/L [20] and 3.65 ± 0.76–5.19 ± 1.00 mmol/L [42] in the period up to t_5_.

#### 3.6.3. Area under the Curve (AUC)

Four studies reported AUC [µmol × h]/L for periods t_0_–t_4_ and t_5_–t_8_ for groups 2 and 3, respectively [20,21,24,42]. The data show that in two studies [21,24], the AUC for the period t_5_–t_8_ are significantly higher in groups 2 and 3 than for the period t_0_–t_4_, whereby the renewed MCT intake at t_4_ should be considered (see Figure 2). Based on the complete data from St-Pierre et al. (2019) [21] and Vandenberghe et al. (2017) [24], the mean AUC for the entire period of 8 h in group 3 is 21.8% higher than the mean AUC in group 2. The AUC of group 1 for the period t_0_–t_4_ was, on average, 10.7% higher than the AUC of group 2 and 50.4% higher than the AUC of group 3. There was a high degree of heterogeneity in the individual data (see Table 5).

#### 3.6.4. Octanoat (C8) and Decanoat (C10)

**Figure 6:**
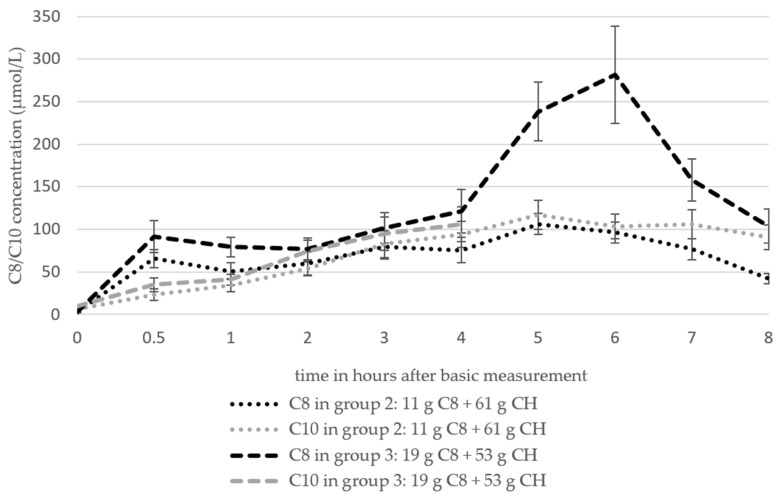
The course of the C8 and C10 concentrations in plasma based on the analysis of the total plasma lipids of groups 2 and 3 (mean values ± standard deviation). The number of studies (n) of the individual measurement time points: group 2 (C8 and C10): t_0_ = 3, t_0,5_ = 3, t_1_ = 3, t_2_ = 3, t_3_ = 3, t_4_ = 3, t_5_ = 1, t_6_ = 1, t_7_ = 1, t_8_ = 1; group 3 (C8): t_0_ = 3, t_0,5_ = 3, t_1_ = 3, t_2_ = 3, t_3_ = 3, t_4_ = 3, t_5_ = 1, t_6_ = 1, t_7_ = 1, T_8_ = 1; group 3 (C10): t_0_ = 2, t_0,5_ = 2, t_1_ = 2, t_2_ = 2, t_3_ = 2, t_4_ = 2.

The data pertaining to the mean plasma C8 and C10 concentrations of groups 2 and 3 (Figure 6) were derived from the studies by Courchesne-Loyer et al. (2017) (emulsion and non-emulsion, considered here as n = 2) [25] and St-Pierre et al. (2019) [21]. The data from the study by Heidt et al. (2023) [42] are listed separately (Table 6), as the free fatty acids were determined rather than the plasma total lipids.

The C8 values of both groups based on the analysis of the total plasma lipids increased from 2.8 ± 0.47 µmol/L (baseline) to 65.41 ± 10.35 µmol/L (group 2) and 91.31 ± 18.5 µmol/L (group 3) by t_0.5_ and increased to 75.59 ± 14.79 µmol/L (group 2) and 121.41 ± 25.36 µmol/L (group 3) by t_4_. The data basis after t_4_ is one study in each group (n = 1). C8 increased in group 2 to 106.17 ± 12.35 µmol/L after 5 h and then fell to a value of 41.98 ± 6.17 µmol/L after 8 h. The values of group 3 reached a maximum value of 281.48 ± 56.79 µmol/L at t_6_ and then also fell to 103.7 ± 19.75 µmol/L. C10 increased in both groups from 6.2 ± 1.13 µmol/L (group 2) and 9.3 ± 1.7 (group 3) to 93.87 ± 15.36 µmol/L (group 2) and 105.94 ± 20.77 µmol/L at t_4_.

In accordance with the values proposed by Heidt et al. (2023) [42], plasma C8 and C10 demonstrated a continuous increase until t_3_ (C8)/t_4_ (C10), after which they exhibited a stagnation until t_5_. The C8 values exhibited a range of 0.00853 ± 0.00038 to 0.1167 ± 0.0572 µmol/L, while the C10 values demonstrated a range of 0.0045 ± 0.00087 to 0.0279 ± 0.0194 µmol/L.

#### 3.6.5. Side Effects of MCT Intake

The occurrence of side effects associated with MCT intake was documented in three of the seven studies (43%) [20,25,42]. In the remaining studies, no information on side effects was provided. No dropouts were reported in four studies (57%) (see Table 2) [22,24,25,42], while in two studies (29%), one participant did not complete all interventions [21] or discontinued the study due to severe diarrhoea [20]. In one study (14%), up to four participants did not complete all interventions [43]. No reasons were given for any of the interventions that were not carried out [21,43]. In the studies by Heidt et al. (2023) [42] and Courchesne-Loyer et al. (2017) [25], detailed information on the side effects was provided. A total of 5 of the 19 participants (26%) in the study by Heidt et al. (2023) [42] experienced side effects. During the MCT + glucose intervention, there were 50% fewer side effects reported compared to the MCT intervention without CH. The incidence of nausea was observed in five cases (26%), only in the MCT intervention without CH. However, the incidence of diarrhoea, stomach discomfort and abdominal pain was identical for both interventions (n = 2 [11%]/n = 4 [21%]/n = 4 [21%]). No side effects were reported in the controls (water [42] or meal without MCT intake [25]) in both surveys. With the MCT non-emulsions (MCT-NE) in the study by Courchesne-Loyer et al. (2017) [25] (n = 10), the most frequently reported side effects were abdominal discomfort and diarrhoea. These were reported on all test days apart from the control. The frequency of these two side effects (ad-dotted) increased in a dose-dependent manner (10 g MCT-NE: n = 2, 20 g MCT-NE: n = 5, 30 g MCT-NE: n = 9). With the MCT emulsions (MCT-EM), the most frequently reported side effect was abdominal discomfort (10 g MCT-EM: n = 2 [20%], 20 g MCT-EM: n = 2 [20%], 30 g MCT-EM: n = 4 [40%]). Diarrhoea did not occur. However, in contrast to the NE interventions, nausea was reported with 20 g MCT-EM (n = 2 [20%]) and with 30 g MCT-EM (n = 1 [10%]). Overall, fewer MCT-typical side effects occurred with MCT-EM than with MCT-NE. In the studies by Heidt et al. (2023) and Courchesne-Loyer et al. (2017), gastrointestinal complaints generally disappeared within 30–60 min after ingestion of the intervention product [25,42]. Table 7 shows the main MCT-associated side effects of the studies by Heidt et al. (2023) [42] and Courchesne-Loyer et al. (2017) [25].

## 4. Discussion

The course of total ketones and ßHB showed that the intake of C8-containing MCT (⌀ 22.5 g C8) without CH intake had the strongest ketogenic effect. In the presence of CH intake, MCT-induced ketogenesis appears to be slightly impaired. This effect is consistent with the observations of an intervention study from 1958, in which the ketone production induced by C8 infusions (500 mL infusion, 1.5% C8) was significantly reduced by a glucose infusion (20 g) and a glucose–insulin infusion (20 g/0.1 unit/kg body weight HGF-free insulin). In the underlying trial, however, only acetone was quantified [48]. In contrast, a study from 1964 showed that C8 oxidation with low quantities of C8 is probably not significantly impaired by a moderate carbohydrate intake (100 g oral glucose/1 g glucose infusion) in the human organism [48], which challenges the aforementioned effect.

Furthermore, the results of the review indicate that an intake of ⌀ 11.4 g C8 (group 2) did not have a significantly lower ketogenic effect than the intake of ⌀ 18.6 g C8 (group 3). As the review is based on different trials with heterogeneous intervention conditions, no definitive statements can be made about the effect. However, the latter intake level corresponds to an additional C8 intake of 63%. Another noteworthy aspect is the higher CH-to-C8-intake ratio observed in group 2 (5.36 g CH/g C8) compared to group 3 (2.87 g CH/g C8). The bias described above, due to the values of St-Pierre et al. (2019) [21], in which the baseline values were subtracted, which were not disclosed, affects groups 2 and 3. Therefore, the comparison of the two groups can be regarded as meaningful. As the ßHB values from the study by Norgren et al. (2020) [20] were also included in the total ketones of group 3, the corresponding curve was adjusted slightly downward due to the absence of AcAc values within the first 4 h. The present results indicate a non-linear increase in ketone levels with increasing C8 intake (see Figure 4B). This finding contrasts with the results of Courchesne-Loyer et al. (2017), who observed a generally linear relationship between MCT intake and ketone production [25]. In a further randomised intervention study from 2023, 10 g, 20 g or 30 g of MCT or a placebo were administered, with the C8 content not specified. The results demonstrated that the ketone concentration in the blood increased in a manner dependent on the MCT intake, with higher MCT intake resulting in elevated βHB and total ketone levels [49]. Bergen et al. (1966) showed a statistically significant increase in the dextrose–MCT intervention from 0.18 mmol/L (t_0_) to 0.78 mmol/L (1.9 mg/100 mL (t_0_) to 8.1 mg/100 mL) in 14 healthy volunteers using 47.3 g dextrose and 100 g MCTs (86% C8, 14% C10) or 100 g corn oil (each + casein and water). The intake of a slightly lower amount of CH with a significantly higher amount of MCT and C8 compared to groups 2 (+75 g C8, 754% of group 2) and 3 (+67 g C8, 462% of group 3) of this review led to almost half as high maximum values (group 2: 0.38 ± 0.08 mmol/L (t_0.5_)/group 3: 0.32 ± 0.09 mmol/L (t_1_)) in the period up to t_3_ [50]. As a result, no linear increase in ketones can be observed with increasing amounts of C8 and CH, whereby an increased amount of C8 led to increased ketones in the study of Bergen et al. (1966) [50]. In a review, Cunnane et al. (2016) again suggested a linear relationship between oral MCT intake up to 70 g/d and a maximum measured plasma ßHB concentration [51]. After reviewing the studies used, Lin et al. (2021) concluded that they were too heterogeneous to make such a statement due to subject groups, doses, previous fasting duration and concurrent meal intake [33]. Lin et al. (2021) criticise the fact that the review by Cunnane et al. (2016) did not focus on C8 and suggests a non-linear relationship starting at relatively low doses [33,51]. This is consistent with the results presented here. In the limited number of studies that have analysed blood glucose levels [20,42,43], a consistent pattern with minor fluctuations without any significant abnormalities has been observed. This consistent pattern has been documented since a survey conducted in 1966, which demonstrated that the type of intervention fat (MCT/corn oil) had no influence on blood sugar levels [50]. The trends in ketone body levels, therefore, do not appear to be directly related to blood glucose.

Only limited data are available for the period following t_4_ (n = 2–3). It is assumed that an additional fasting effect could occur, namely a physiological increase in ketones, which could support ketone production. It is unclear why the ketone level of group 3 does not clearly show this effect. The results of the influence of the interventions on the AUC demonstrate a similar effect in groups 2 and 3. The AUC was significantly higher from t_4_–t_8_ than in the period t_0_–t_4_. It should be noted that in three of the underlying studies, a further MCT intake was added at t_4_ without a new CH intake (see Figure 2). A comparison of the mean values revealed that the AUC of the MCT group without CH intake was higher than in the groups with CH intake. This finding is consistent with the above observation on the course of total ketones with/without CH. The AUC appears to be useful for assessing the change over the total time within a study. The value of inter-study comparisons is limited due to differences in calculation bases.

The plasma C8 and plasma C10 curves (Figure 6) show a relatively parallel course to the total ketones (Figure 4A) with a slight shift of the graphs to the left. It can, therefore, be assumed that the body successfully converts the MCFA from the blood into ketones. In another survey from 2018, in which MCTs were supplied in a meal-like intervention, the MCFA also showed a similar kinetic profile to the ketones [52]. Against this background, the pronounced increase and subsequent decrease in C8 in group 3 between 4 and 8 h after the intervention is conspicuous (Figure 6). However, the effect is based on a single study (n = 1) in which the course of the total ketones coincides with the course of C8 [21]. In the present evaluation, the data from two studies [21,25] were initially considered, in which the plasma MCFAs were analysed on the basis of the plasma total lipids. In a further survey by Heidt et al. (2023) [42], the free fatty acids were quantified. After the conversion of the data from Heidt et al. into µmol/L, a comparison of the two measurement methods revealed a significant difference in the measured values. The measured values of the first two studies (group 3) were notably higher than those observed in the study by Heidt et al. (2023) (C8: 16–281 µmol/L [21,25] vs. 0.09–0.12 µmol/L [42]; C10: 5–147 µmol/L [21,25] vs. 0.005–0.03 µmol/L [42]). This effect can be attributed to the comparison of protein-bound fatty acids with plasma total lipids. The graphs of the two measurement methods (group 3) in the trials demonstrate a comparable course over the 4 h period following the intervention [21,25,42]. St-Pierre et al. (2019) critique the analysis of total plasma lipids, citing uncertainty regarding the proportion of MCFA present in the free fatty acid form or in small proportions in the esterified form [21]. Furthermore, interference with other lipids could occur, potentially complicating the interpretation of the results. However, the method has the advantage of encompassing the entire pool of fatty acids. Interference with other free fatty acids could also occur when measuring free fatty acids. Overall, it is unclear to what extent MCTs are present in the organism as triglycerides, e.g., in plasma, after temporary or permanent ingestion. It is known that MCFAs, in contrast to long-chain fatty acids (LCFA), are not easily esterified and incorporated into lipids, as their ability to bind to fatty acid-binding protein is limited [53]. In addition, MCTs are only incorporated in trace amounts as liver fat [54].

The data from all three groups were included in the analysis, regardless of the subjects’ caffeine intake and the extent of dispersion. The conditions within the groups 1 and 2 were relatively homogeneous. In group 1, mixtures were added uniformly, while in one of the three studies, caffeine was added. In group 2, no caffeine was used uniformly, and emulsions were used in four of the five included studies. The degree of dispersion in group 3 is more heterogeneous, with three emulsions and three mixtures. Consequently, while the groups themselves are relatively homogeneous, the type of dispersion is not uniform when compared with each other. As the survey by Courchesne-Loyer et al. (2017) [25] demonstrates, emulsions promote ketone production. The observed effect that an emulsified MCT intake supports ketosis in a dose-dependent manner, while a non-emulsified intervention has a dose-independent effect, is likely attributable to the fact that the local maximum absorption is reached by the emulsion. Non-emulsified MCTs are probably absorbed at a slower rate and with reduced efficiency. It may, therefore, be postulated that the dose-independent effect is due to the fact that the maximum absorption is not reached as efficiently as with emulsified MCTs [25]. Since the majority of the studies from group 2 used emulsions, this could have shifted the ketone level upwards compared to groups 1 and 3. A study by Vandenberghe et al. (2017) [55] also demonstrated that caffeine significantly increased ketone production (+88%/+116%) and free fatty acids in plasma in a dose-dependent manner (2.5/5.0 mg/kg body weight) despite the intake of a standard breakfast (85 g CH, 9.5 g fat, 14 g protein). Another study by Bellet et al. (1968) [56] highlighted the beneficial impact of caffeine (250 mg) on free fatty acids. It is known from another study that an intervention of caffeine and MCTs (C8) together has a stronger ketogenic effect than MCTs (C8) without caffeine, as well as an intervention without MCTs (C8) or caffeine. However, βHB was measured in capillary blood, and no CH intake was provided [57]. Due to the intake of caffeine in the study by Norgren et al. (2020) [20], it can, therefore, be assumed that the ketone curves of groups 1 and 3 were shifted upwards. With regard to group comparability, it should be noted that the non-uniform dispersion type and caffeine intake across groups represent an influencing factor on ketone production. In addition to the C8 amounts considered, the MCT intervention amounts differed between the three groups (19.2–41.0 g). While C12 was slightly present in all groups (0.0–0.4 g), groups 2 (7.6 ± 0.5 g) and 3 (5.0 ± 6.1 g) had lower amounts of C10 compared to group 1 (18.1 ± 17.4 g) (see Appendix B). It is well established that the ketogenic effect of C8 in the human organism is significantly stronger than that of C10 [24] (approx. three times [21]), so the influence of C10 on the results can be classified as low when the intervention quantities are taken into account.

The most commonly observed MCT-specific side effects include flatulence, diarrhoea, abdominal cramps, nausea and vomiting [57,58,59,60,61,62]. In the studies by Heidt et al. (2023) [42] and Courchesne-Loyer et al. (2017) [25], clear, mainly gastrointestinal side effects were reported. Consequently, the non-reporting of side effects in some studies (n = 4) indicates a lack of information and not necessarily the absence of side effects. The small number of dropouts in the included trials suggests that there were tolerable and no serious side effects. The result that the side effects dissipated within 30–60 min, i.e., can be considered temporary overall [25,42], supports this assumption. A study from 2021, in which 0–42 g MCTs (99% C8) were administered, found that all symptoms disappeared completely a few minutes after eating a meal [63], confirming this hypothesis. In the included studies, side effects also occurred in principle during an intervention with a meal or CH intake [25,42]. Despite a reduction in the total number of side effects observed in the study by Heidt et al. following the intake of CH compared to the intervention without CH, the occurrence of abdominal and intestinal side effects was found to be similar in both groups [42]. This finding challenges the prevailing opinion, which suggests an increase in the tolerability of MCTs when consumed with a meal [42,64,65], whereby the described effect is frequently observed in clinical practice. Of the respective underlying sources [66,67], only Henderson et al. (2009) [66] report a significant decrease in the severity and dropouts due to gastrointestinal side effects after the MCT application was switched from an MCT drink to a liquid meal replacement due to side effects during the course of the study. The tolerability of MCTs appears to vary considerably between individuals, with the frequency of side effects appearing to be dose-dependent on MCT intake [25,42]. Emulsification appears to reduce side effects, particularly diarrhoea [25]. In a survey by Xu et al. (2022), a decrease in symptoms (diarrhoea) was observed with increasing intervention duration at constant MCT quantities [61]. This indicates a habituation effect of MCT.

In this review, a caloric density of 8.3 kcal per gram of MCT was used to calculate MCT-related caloric intake based on market research. The underlying sources [27,31], which give a range of 8.3–8.4 kcal/g MCT, each refer to an article by Ingle et al. (1999) [68], which, however, advocates a net energy value of 6.80 kcal/g MCT. The value was derived on the basis of animal studies with different MCT compositions [68]. Human studies also suggest a lower energy content [69,70], but the exact energy content of MCTs is currently unknown. Commercially available products often use the usual 9 kcal/g corresponding to long-chain fatty acids according to the Food Information to Consumers Regulation (FIC Regulation) [71].

The number of studies investigating the influence of CH on C8-induced ketogenesis is very limited. With a total of seven studies of different research designs, there are few, and also very heterogeneous surveys, which indicates an existing need for research. In line with the results of the Rob II tool, the quality of the studies—excluding the aspect of heterogeneity—can be considered acceptable overall, whereby the lack of breakdown of the total ketones in ßHB and AcAc is one of the critical points worth mentioning. Within the studies, different interventions with varying nutrient profiles were provided with standard breakfasts and drinks, making it difficult to compare the individual studies with each other. For this reason, groups were formed to make the individual interventions comparable in total. The groups do not represent a standardised study procedure but merely a division according to the amount of C8 and CH used. Despite the limitations mentioned, this systematic review forms an essential basis for further studies and clearly shows that greater standardisation and better study quality are required in the future.

## 5. Conclusions

Overall, the data available up to 4 h after the start of the intervention (t_4_) can be considered sufficient (n = 3–6) to show trends in the influence of CH intake on MCT-induced ketogenesis. Ketone production appears to be slightly decreased in interventions containing C8 and a source of CH compared to MCT intake alone. A linearity between increased C8 and CH intake and rising ketone levels could not be determined, whereby the heterogeneous intervention conditions between the studies minimise the validity of the results. As linear developments have been reported sporadically in the literature, there is a need for research to clarify the specific influence of different amounts of C8 in combination with CH on ketogenesis. The meta-analytical analysis revealed a high level of heterogeneity in the data, which is considered a critical issue and raises further questions in the field of study. Finally, further studies under homogeneous conditions are required to determine the influence of different CH forms and complex meals under the influence of C8 on ketogenesis to investigate the flexibility in the implementation of a KD with the addition of C8-containing MCT. Other C8 and CH doses should also be tested in addition to the doses used to date.

## Figures and Tables

**Table 1:** Inclusion and exclusion criteria of the systematic literature review, taking into account the PICO scheme [38].

Unit	Inclusion Criteria	Exclusion Criteria
Population	Healthy people (regardless of age, gender, ethnicity)	In vitro studies, animal studies, existing diseases (e.g., diabetes mellitus, insulin resistance, metabolic diseases, epilepsy), people on a KD, professional athletes
Intervention	Dietary intervention with intake or complementation of MCT- or MCFA-containing products with C8 content with simultaneous oral CH intake	Intervention without MCT or MCFA supply or with supply of exogenous ketone bodies, no accompanying use of CH
Comparison	Completed and published studies, controlled and uncontrolled, as well as randomised and non-randomised intervention studies	Ongoing and unpublished studies, poster abstracts, observational studies, reviews, meta-analyses
Outcome	Essential: ßHB (plasma ßHB or total ketones (ßHB and AcAc)); optional: acetoacetate, acetone, plasma C8/C10/C12, plasma glucose, AUC ^1^ ßHB/total ketones, side effects	Ketone concentration measured only in the form of acetone or acetoacetate

^1^ AUC = area under the curve.

**Table 2:** Characteristics of the included studies with focus on baseline and methodological aspects.

Author, Year, Country	Age (Ø Years) m/f (%)	N	Blinding	Dropout Rate (%)
**RCT**				
Courchesne-Loyer et al. (2017) [25] Canada	31.0 ± 3.0 60/40	10	Single	0
Norgren et al. (2020) [20] Sweden	69.2 ± 2.4 47/53	15	Single	One subject (n = 1) dropped out and was replaced by another from the waiting list. This participant went through all intervention arms.
St-Pierre et al. (2019) [21] Canada	34.0 ± 12.0 78/22	9	Single	One subject (n = 1) did not take part in one of the interventions. The available data were analysed.
Vandenberghe et al. (2017) [24] Canada,	34.0 ± 12.0 78/22	9	Single	0
**Controlled cross-over study**				
Heidt et al. (2023) [42] Germany	25.0 (20.0–27.0) 37/63	19	Single	0
**Controlled intervention study**				
Vandenberghe et al. (2020) [43] Canada	28.0 ± 7.0 ^1^ 40/60 ^1^	10 ^1^	-	Four subjects (n = 4) did not carry out all interventions. The available data were analysed.
**Uncontrolled intervention study**				
Freemantle et al. (2009) [22] Canada	48.0 ± 1.3 -/-	32	-	0

^1^ Another age group was analysed in this study. The underlying results were not included in this study, so the additional age group is not presented.

**Table 3:** Characteristics of the interventions considered in the studies, including nutrient composition.

Study	Intervention		Nutrient Composition	Measurement Parameters Considered
	Point of Time	Components		
	Within 15 min after the basic measurement	Standard breakfast: 2 slices of toast, jam +		
		1. 280 mL lactose-free, low-fat milk, 12 g C8, 8 g C10 (emulsion)	**Carbohydrates: 64.5 g** Protein: 14.3 g	
Courchesne-Loyer et al. (2017) [25]		2. 280 mL lactose-free, low-fat milk, 12 g C8, 8 g C10 (non-emulsion) 3. 270 mL lactose-free, low-fat milk, 18 g C8, 12 g C10 (emulsion) 4. 270 mL lactose-free, low-fat milk, 18 g C8, 12 g C10 (non-emulsion)	Fat: 26.4 g Total MCT: 20.0 g ^2^ **C8: 12.0 g** C10: 8.0 g Energy: 544.2 kcal **Carbohydrates: 64.2 g** Protein: 13.9 g Fat: 36.2 g Total MCT: 30.0 g ^2^ **C8: 18.0 g** C10: 12.0 g Energy: 623.5 kcal	Total ketones ^1^, AUC (ßHB), Plasma C8/10/12
Freemantle et al. (2009) [22]	Within 30 min after the baseline measurement	Ketogenic breakfast drink: 100 g cream, 25 g protein powder (milk), 46 g water, 71 g MCTs	**Carbohydrates: 3.0 g** Protein: 25.0 g Fat: 110.0 g Total MCT: 71.0 g ^2^ **C8: 28.3 g** C10: 41.6 g C12: 1.1 g Energy: 1.012.0 kcal	Plasma glucose, ßHB
Heidt et al. (2023) [42]	< 1 min after the baseline measurement	1. 200 mL water, 0.5 g/kg KG MCT oil 2. 200 mL water, 0.5 g/kg KG MCT oil, 0.2 g/kg KG glucose On average: 32 (30.0–35.5) g MCTs (19.2 g C8, 12.8 g C10) 12.7 (11.8–13.8) g Glucose	**Carbohydrates: 0.0 g** Protein: 0.0 g Fat: 32.0 g Total MCT: 32.0 g ^2^ **C8: 19.2 g** C10: 12.8 g Energy: 266.0 kcal **Carbohydrates: 12.7 g** Protein: 0.0 g Fat: 32.0 g Total MCT: 32.0 g ^2^ **C8: 19.2 g** C10: 12.8 g Energy: 317.5 kcal	Plasma glucose, ßHB, total ketones ^1^, AUC (ßHB), plasma C8/C10
Norgren et al. (2020) [20]	Within 5–7 min after the basic measurement	2.5 dL coffee (approx. 170 mg caffeine), 15 g cream + 1. 10 g sunflower oil + 20 g C8 2. 10 g sunflower oil + 20 g C8 + 50 g glucose	**Carbohydrates: 1.2 g** Protein: 0.9 g Fat: 34.8 g Total MCT: 20.0 g ^2^ **C8: 20.0 g** Energy: 305.6 kcal **Carbohydrates: 51.1 g** Protein: 0.9 g Fat: 34.8 g Total MCT: 20.0 g ^2^ **C8: 20.0 g** Energy: 508.4 kcal	Plasma glucose, ßHB, AUC (ßHB)
St-Pierre et al. (2019) [21]	Breakfast after the baseline measurement, test drink after the baseline measurement and 4 h (t_4_) after the baseline measurement	Standard breakfast: 2 slices of toast, raspberry jam, 1 piece of cheese, 2 scrambled eggs + 1. 250 mL lactose-free, low-fat milk + 17.3 g C8, 1.0 g C12 2. 250 mL lactose-free, low-fat milk + 9.5 g C8, 6.7 g C10, 1.7 g C12	**Carbohydrates: 62.0 g** Protein: 32.5 g Fat: 41.5 g Total MCT: 18.2 g ^2^ **C8: 17.3 g** C12: 1.0 g Energy: 718.1 kcal **Carbohydrates: 62.0 g** Protein: 32.5 g Fat: 41.2 g Total MCT: 17.9 g ^2^ **C8: 9.5 g** C10: 6.7 g C12: 1.7 g Energy: 715.6 kcal	ßHB, total ketones ^1^, AUC (ßHB), plasma C8/C10/C12
Vandenberghe et al. (2017) [24]	Breakfast after the baseline measurement, test drink after the baseline measurement and 4 h (t_4_) after the baseline measurement	Standard breakfast: 2 slices of toast, jam, 1 piece of cheese, 2 scrambled eggs + 1. 250 mL lactose-free, low-fat milk + 11.4 g C8 & 7.6 g C10 2. 250 mL lactose-free, low-fat milk + 19.0 g C8	**Carbohydrates: 65.4 g** Protein: 34.9 g Fat: 44.6 g Total MCT: 19.0 g ^2^ **C8: 11.4 g** C10: 7.6 g Energy: 794.6 kcal **Carbohydrates: 65.4 g** Protein: 34.9 g Fat: 44.6 g Total MCT: 19.0 g ^2^ **C8: 19.0 g** Energy: 794.6 kcal	Total ketones ^1^, AUC (ßHB)
Vandenberghe et al. (2020) [43]	Breakfast after the baseline measurement, test drink after the baseline measurement and 4 h after the baseline measurement (t_4_)	Standard breakfast: 2 slices of wholegrain toast, strawberry jam, 1 slice of cheese, 2 scrambled eggs + 100 mL water, emulsifier, sweetener, 12 g C8, 8 g C10	**Carbohydrates: 49.0 g** Protein: 23.2 g Fat: 39.5 g Total MCT: 20.0 g ^2^ **C8: 12.0 g** C10: 8.0 g Energy: 626.0 kcal	Plasma glucose, total ketones ^1^, AUC (ßHB)

^1^ Total ketones = ßHB + AcAc. ^2^ MCT contents correspond to the added MCT quantities.

**Table 4:** Caffeine intake and dispersion type of MCT intake in groups 1 to 3.

Study	Caffeine Intake	Dispersion Type
**Group 1: 23 g C8 without CH**		
Freemantle et al. (2009) [22]	No	Mixture
Heidt et al. (2023) [42]	No	Mixture
Norgren et al. (2020) [20]	Yes	Mixture
**Group 2: 11 g C8 + 61 g CH**		
Courchesne-Loyer et al. (2017) [25]	No	Emulsion
Courchesne-Loyer et al. (2017) [25]	No	Mixture
St-Pierre et al. (2019) [21]	No	Emulsion
Vandenberghe et al. (2020) [43]	No	Emulsion
Vandenberghe et al. (2017) [24]	No	Emulsion
**Group 3: 19 g C8 + 53 g CH**		
Courchesne-Loyer et al. (2017) [25]	No	Emulsion
Courchesne-Loyer et al. (2017) [25]	No	Mixture
Heidt et al. (2023) [42]	No	Mixture
Norgren et al. (2020) [20]	Yes	Mixture
St-Pierre et al. (2019) [21]	No	Emulsion
Vandenberghe et al. (2017) [24]	No	Emulsion

**Table 5:** AUC (ßHB or ßHB plus AcAc) for the periods t_0_–t_4_ and t_5_–t_8_ of groups 2 and 3.

	23 g C8 without CH (Group 1)		11 g C8 + 61 g CH (Group 2)		19 g C8 + 53 g CH (Group 3)	
	AUC t_0_–t_4_ [µmol × h]/L	AUC t_5_–t_8_ [µmol × h]/L	AUC t_0_–t_4_ [µmol × h]/L	AUC t_5_–t_8_ [µmol × h]/L	AUC t_0_–t_4_ [µmol × h]/L	AUC t_5_–t_8_ [µmol × h]/L
Heidt et al. (2023) [42] (ßHB) ^1^	1022.37 ± 331.18	-	1033.92 ± 403.35	-	-	-
Norgren et al. (2020) [20] (ßHB)	450.00 ± 190.00	-	-	-	280.00 ± 120.00	-
St-Pierre et al. (2019) [21] (ßHB + AcAc)	-	-	339.29 ± 65.47	1005.95 ± 89.29	407.74 ± 83.33	1217.26 ± 127.97
**Vandenberghe et al. (2017)** [24] **(ßHB + AcAc)**	-	-	621.95 ± 97.56	1548.78 ± 134.15	780.49 ± 152.44	1878.05 ± 274.39
Mean value ± standard deviation	736.19 ± 286.24	-	665.05 ± 285.18	1277.37 ± 271.42	489.41 ± 212.47	1547.66 ± 330.40

^1^ Heidt et al.: t_0_–t_5_.

**Table 6:** The course of the C8 and C10 concentrations in plasma based on the analysis of free fatty acids of group 3 (mean values ± standard deviation) [42].

	t_0_	t_0.5_	t_1_	t_2_	t_3_	t_4_	t_5_
**C8 (µmol/L)**	0.00853 ± 0.00038	0.0341 ± 0.0385	0.0742 ± 0.0711	0.1102 ± 0.0672	0.1167 ± 0.0572	0.1153 ± 0.0591	0.1148 ± 0.0535
**C10 (µmol/L)**	0.0045 ± 0.00087	0.009 ± 0.0105	0.0099 ± 0.0078	0.0197 ± 0.0149	0.0233 ± 0.0193	0.0279 ± 0.0194	0.0278 ± 0.0176

**Table 7:** Overview of main MCT-associated side effects based on two studies (n = 2) [25,42].

	Intervention	Dropout Rate (%)	Nausea (%)	Gastric Reflux (%)	Vomiting (%)	Diarrhoea (%)	Stomach Discomfort (%)	Abdominal Discomfort/Pain (%)
**Courchesne-Loyer et al. (2017) [25]**	MCT emulsion with CH	0	20 (20 g) ^1^ 10 (30 g) ^1^	10 (20 g)^1^	0	0	0	20 (10 g) ^1^ 20 (20 g) ^1^ 40 (30 g) ^1^
	MCT non-emulsion with CH	0	0	10 (30 g)^1^	0	10 (10 g) ^1^ 20 (20 g) ^1^ 50 (30 g) ^1^	0	10 (10g) ^1^ 30 (20 g) ^1^ 40 (30 g) ^1^
	Control (meal without MCT intake)	0	0	0	0	0	0	0
**Heidt et al. (2023) [42]**	MCT with glucose	0	0	0	0	11	21	21
	MCT	0	26	0	5	11	21	21
	Control (water)	0	0	0	0	0	0	0

^1^ Quantities in brackets (g) pertain to the MCT quantity.

## Data Availability

No new data were created or analysed in this study. Data sharing is not applicable to this article.

## Appendix A

**Table A1:** Complete search strategy for the PubMed and Web of Science databases.

Data Base	Search incl. Filter
PubMed	((“carbohydrat*”[Text Word] OR “carbohydrate intake”[Text Word] OR “carbohydrate supply”[Text Word] OR “glucos*”[Text Word] OR “dextros*”[Text Word] OR “sugar*”[Text Word] OR “Carbohydrates”[MeSH Terms] OR “Sugars”[MeSH Terms]) AND (“MCT”[Text Word] OR “mct s*”[Text Word] OR “medium chain triglycerid*”[Text Word] OR “medium chain triglycerid*”[Text Word] OR “caprylic acid*”[Text Word] OR “capric acid*”[Text Word] OR “octanoic acid*”[Text Word] OR “decanoic acid*”[Text Word] OR “Triglycerides”[MeSH Terms] OR “Fatty Acids”[MeSH Terms]) AND (“ketogen*”[Text Word] OR “ketogenic diet”[Text Word] OR “Ketone Bodies”[Text Word] OR “low carb*”[Text Word] OR “low carb*”[Text Word] OR “beta-hydroxybutyrate”[Text Word] OR “Ketone Bodies”[MeSH Terms] OR “diet, carbohydrate restricted”[MeSH Terms])) AND ((casereports[Filter] OR clinicalstudy[Filter] OR clinicaltrial[Filter] OR clinicaltrialphasei[Filter] OR clinicaltrialphaseii[Filter] OR clinicaltrialphaseiii[Filter] OR clinicaltrialphaseiv[Filter] OR clinicaltrialprotocol[Filter] OR comparativestudy[Filter] OR controlledclinicaltrial[Filter] OR multicenterstudy[Filter] OR observationalstudy[Filter] OR pragmaticclinicaltrial[Filter] OR randomizedcontrolledtrial[Filter] OR twinstudy[Filter] OR validationstudy[Filter]) AND (humans[Filter]) AND (english[Filter]))
Web of Science	Carbohydrat* OR “carbohydrate intake” OR “carbohydrate supply” OR glucos* OR dextros* OR sugar* OR sucros* (Topic) and MCT OR MCTs* OR “medium-chain triglycerid*” OR “medium chain triglycerid*“ OR “caprylic acid*” OR “capric acid*” OR “octanoic acid*” OR “decanoic acid*” (Topic) and ketogen* OR “ketogenic diet” OR “ketone bodies” OR “low-carb*” OR “low carb*” OR “beta-hydroxybutyrate” OR “carbohydrate restricted*” OR “carbohydrate-restricted” (Topic) and Preprint Citation Index (Exclude–Database) and English (Languages) and Article or Other or Dissertation Thesis or Clinical Trial or Abstract or Meeting or Case Report or Unspecified or Early Access (Document Types)

## Appendix B

**Table A2:** Characterisation of the three intervention groups.

	Group 1	Group 2	Group 3
Studies from which intervention arms were taken	Freemantle et al. (2009) [22] Heidt et al. (2023) [42] Norgren et al. (2020) [20]	Courchesne-Loyer et al. (2017) [25] St-Pierre et al. (2019) [21] Vandenberghe et al. (2017) [24] Vandenberghe et al. (2020) [43]	Courchesne-Loyer et al. (2017) [25] Heidt et al. (2023) [42] Norgren et al. (2020) [20] St-Pierre et al. (2019) [21] Vandenberghe et al. (2017) [24]
Interventions: CH (⌀) (mean values ± standard deviation)	1.4 ± 1.2 g	61.1 ± 6.1 g	53.3 ± 18.8 g
Interventions: MCT (⌀) (mean values ± standard deviation)	C8: 22.5 ± 4.1 g C10: 18.1 ± 17.4 g C12: 0.4 ± 0.5 g MCT: 41.0 ± 21.8 g	C8: 11.4 ± 1.0 g C10: 7.6 ± 0.5 g C12: 0.4 ± 0.7 g MCT: 19.2 ± 0.9 g	C8: 18.6 ± 0.9 g C10: 5.0 ± 6.1 g C12: 0.2 ± 0.4 g MCT: 23.8 ± 5.9 g
Ratio g CH/g C8		5.36	2.87
